# Erratum

**Published:** 1850-08

**Authors:** 


### ERRATUM.

The word *spasmodic*, in the third line of Dr. Slusser's article on Nitrate of Silver, should read *sporadic*.

